# In-Depth Analysis of the Extracorporeal Proteome Adsorbed to Dialysis Membranes during Hemodialysis

**DOI:** 10.3390/membranes12111120

**Published:** 2022-11-09

**Authors:** Lisa Daniel-Fischer, Isabel J. Sobieszek, Anja Wagner, Juan Manuel Sacnun, Bruno Watschinger, Christoph Aufricht, Klaus Kratochwill, Rebecca Herzog

**Affiliations:** 1 Division of Pediatric Nephrology and Gastroenterology, Department of Pediatrics and Adolescent Medicine, Comprehensive Center for Pediatrics, Medical University of Vienna, 1090 Vienna, Austria; 2 Christian Doppler Laboratory for Molecular Stress Research in Peritoneal Dialysis, Medical University of Vienna, 1090 Vienna, Austria; 3 Division of Nephrology and Dialysis, Department of Inner Medicine III, Medical University of Vienna, 1090 Vienna, Austria

**Keywords:** hemodialyzer, protein loss, protein adsorption, proteome of hemodialyzer membranes

## Abstract

Used hemodialysis membranes (HD-M) are a valuable reservoir of biological information. Proteins bind to HD-M, but whether this process depends on the type of membrane or patient factors or selectively affects specific protein classes has not been adequately elucidated. State-of-the-art proteomics techniques are capable of identifying and quantifying this therapy-specific subproteome to enable the analysis of disease- or membrane-induced pathophysiologies. We demonstrate the feasibility of the deep proteomic characterization of the extracorporeal proteome adsorbed to HD-M. A shotgun proteomics approach using nano-flow liquid chromatography coupled to mass-spectrometry identified 1648 unique proteins eluted by a chaotropic buffer from the HD-M of eight patients. In total, 995 proteins were present in all eluates; a more stringent approach showed that a core proteome of 310 proteins could be identified independently in all samples. Stability of the dialyzer proteome was demonstrated by a >90% re-identification rate on longitudinal samples of a single patient. The core proteome showed an overrepresentation of pathways of hemostasis and the immune system, and showed differences in membrane materials (polysulfone vs. helixone). This study demonstrates that optimized conditions combined with high-performance proteomics enable the in-depth exploration of the subproteome bound to HD-M, yielding a stable core proteome that can be exploited to study patient-specific factors and improve hemodialysis therapy.

## 1. Introduction

End-stage kidney disease (ESKD) affects millions of patients of all ages, with increasing prevalence worldwide. Kidney transplantation, as the best treatment option in terms of survival, quality of life, and total cost, is limited by donor organs. The other options of renal replacement therapy are hemodialysis (HD) and peritoneal dialysis (PD), with HD being the most commonly used modality [1]. Renal replacement therapies aim to remove excess fluid, metabolic waste products, and uremic toxins from patients while retaining other solutes such as nutrients and proteins in the body.

Protein loss represents one of the major clinical problems during renal replacement therapy with dialysis [2]. The lost proteins need to be substituted by metabolic processes, and in turn generate uremic toxins, which again need to be removed by dialysis [3]. The unintentionally removed plasma proteins are important variables in determining the biocompatibility of the dialysis therapy [4,5,6]. The systemic effect of this protein loss is not yet sufficiently understood.

Used dialyzers, normally discarded as a waste product of HD treatment, are recognized as repositories of relevant biological and dialysis technique information [7,8,9,10]. Hemodialyzers are also employed in septic shock as an extracorporeal therapeutic approach for cytokine removal [11,12,13]. The characterization of proteins and cytokines adsorbed to membranes therefore is of high interest but still sparse [6,14,15,16]. To date, attention has been limited to the characterization of protein adsorption with respect to different dialyzers/membrane types [6,9,14]. Modern high-performance proteomics methods potentially enable the in-depth characterization of the adsorbed proteins [10].

Here, we aimed to identify proteins bound to the hemodialyzer membranes of patients with ESKD. The description of the extracorporeal proteome of patients with different underlying diseases or therapy regimens could provide insight into the mode of action of the therapy, related pathomechanisms, or side effects, and it could lead to the development of more biocompatible dialyzer membranes and yield biomarkers for the improved management of dialysis therapy.

## 2. Materials and Methods

Standard chemicals were obtained from Sigma-Aldrich (St. Louis, MO, USA), unless otherwise specified.

### 2.1. Sample Collection

Eleven dialyzers were collected from eight different anonymized ESKD patients undergoing regular (3 to 4 times per week) treatments on standard HD regimens for at least 3 months. All patients were dialyzed with polysulfone-based membranes. Four of the patients were dialyzed with helixone membranes (Fx100, effective surface area 2.2 m^2^, Fresenius Medical Care, Bad Homburg, Germany) and 4 patients with classical polysulfone membrane dialyzers (Leoceed-21H, effective surface area 2.1 m^2^, Asahi Medical Co., Tokyo, Japan; Diacap Pro 19H, effective surface area 1.9 m^2^, B. Braun Avitum Medical Inc., Melsungen, Germany). Helixone is a synthetic polysulfone-based, high-flux membrane characterized by nanoscale modulation of the innermost surface structures [6,17]. For the reproducibility experiment, 4 dialyzers of 1 patient were collected within 10 days (day 1, 3, 5, 9). The study was approved by the local ethics committee of the Medical University of Vienna (EK 1925/2020). The experiments were performed in accordance with the Declaration of Helsinki 2013.

### 2.2. Protein Elution and Quantification

At the end of the HD session, dialyzers were dismounted according to clinical practice and transferred immediately to the laboratory. The dialyzers were connected to fresh tubing systems and flushed with sterile saline solution to remove any visible residual blood. Subsequently, they were rinsed with 3 L of 0.9% NaCl solution over a period of 30 min before the elution steps. The membrane-adsorbed proteins were eluted by forcing a strong chaotropic buffer (6 M urea, 2 M thiourea, 0.1% sodium dodecyl sulfate (SDS)), supplemented with 1 tablet of Complete Protease Inhibitor (Roche, Basel; Switzerland) and 1 tablet of phosphatase inhibitor (PhosSTOP, Roche, Basel, Switzerland) per 250 mL, and a reducing agent, 1 mM dithiothreitol (DTT) (pH 8–8.5), through the dialyzers in a closed tubing system with a peristaltic pump, a modified protocol based on a previously described workflow [14]. Then, 125 mL of solubilization buffer was introduced in the system and allowed to circulate for 1 hour at a flow rate of 15 mL/min. Each step was performed at room temperature. Dialyzer protein extracts were stored at −80 °C until analysis. The total protein content in the extracts was measured using a Pierce BCA Protein Assay Kit (Thermo Fisher Scientific, Waltham, MA, USA) as per the manufacturer’s instructions.

### 2.3. Protein Digestion and Cleanup

Sample volumes corresponding to 25 µg of total protein of each sample were digested using single-pot, solid-phase enhanced sample preparation (SP3). Briefly, the reduced (10 mM DTT for 1 h at 56 °C) and alkylated (55 mM iodoacetamide (IAA) for 30 min at RT) proteins were bound to SP3 beads (10:1 bead:protein ratio, GE Healthcare, Chicago, IL, USA), washed with 80% ethanol and acetonitrile, and subjected to on-bead digestion with trypsin/LysC Mix (1:25 protease:protein ratio, Promega, Madison, WI, USA) overnight at 37 °C in 50 mM ammonium bicarbonate, pH 8.5. After elution, peptides were desalted using Pierce Peptide Desalting Spin Columns (Thermo Fisher Scientific, Waltham, MA, USA) according to the manufacturer’s instructions. The eluates were dried in a vacuum concentrator and reconstituted in 0.1% trifluoroacetic acid.

### 2.4. Liquid Chromatography–Mass Spectrometry (LC–MS)

Samples were analyzed on an Ultimate 3000 RSLC nano coupled directly to an Orbitrap Fusion Lumos (both Thermo Fisher Scientific) (HD 1-5). Samples were injected onto a reversed-phase C18 column and eluted with a gradient of 4% to 36% mobile phase B over 94 min by applying a flow rate of 300 nL/min. MS scans were performed in the range of *m*/*z* 375–1650 at a resolution of 120,000 (at *m*/*z* = 200). MS/MS scans for peptide identification were performed choosing a top 10 method with a resolution of 15,000; normalized collision energy of 30%; isolation width of 1.6 *m*/*z*; and dynamic exclusion of 90 s. HD 6-11 were analyzed on an Ultimate 3000 RSLC nano coupled directly to an Orbitrap Exploris 480 with a High-Field Asymmetric Waveform Ion Mobility Spectrometry System (FAIMSpro) (all Thermo Fisher Scientific). Samples were injected onto a reversed-phase C18 column (50 cm × 75 µm i.d., packed in-house) and eluted with a gradient of 4% to 38% mobile phase B over 94 min by applying a flow rate of 230 nL/min. MS scans were performed in the range of *m*/*z* 375–1650 at a resolution of 60,000 (at *m*/*z* = 200). MS/MS scans were performed choosing a resolution of 15,000; normalized collision energy of 30%; isolation width of 1.4 *m*/*z*; and dynamic exclusion of 90 s. Two different FAIMS compensation voltages were applied (−40 V and −60 V), with a cycle time of 1.5 s per voltage. FAIMS was operated in standard resolution mode with a static carrier gas flow of 4.1 L/min.

### 2.5. Mass Spectrometry Data Analysis

The acquired raw MS data files were processed and analyzed using Proteome Discoverer (v2.4.0.305, Thermo Fisher Scientific). SequestHT was used as a search engine and the following parameters were chosen: database: Homo sapiens (SwissProt, downloaded on 2019-09-24); enzyme: trypsin; max. missed cleavage sites: 2; static modifications: carbamidomethyl (C); dynamic modifications: oxidation (M), acetyl (protein N-terminus), met-loss (M), met-loss + acetyl (M); precursor mass tolerance: 10 ppm; fragment mass tolerance: 0.02 Da. Precursor ion quantification was performed using the Minora Feature Detector node (Thermo Fisher Scientific, Waltham, MA, USA). Retention time alignment was performed with a maximum RT shift of 10 min and a mass tolerance of 10 ppm. For feature linking, the RT and mass tolerance were set to 0 with a minimum S/N threshold of 5. Only unique peptides were used for intensity-based quantification. Normalization was performed on total peptide amount and scaling mode on all average. Only peptides and proteins with FDR value < 0.01 were reported and single peptide identifications were excluded from the dataset.

### 2.6. Biological Pathway Enrichment Analysis

Functional analysis of identified proteins was performed using Protein Analysis Through Evolutionary Relationships (PANTHER) version 17.0, accessed on 13 July 2022. The PANTHER analysis tool was used to perform enrichment analysis for the identification of over-represented biological terms by a protein list. Annotation databases included in the analysis were the Gene Ontology (GO) cellular component and molecular function, accessed on 13 July 2022. Terms significantly enriched less than 2-fold (Benjamini–Hochberg-corrected *p*-value < 0.01) were excluded. Terms were summarized in a tree map using the rrvgo package in R [18]. Pathway enrichment analysis was performed with the Reactome pathway knowledgebase.

### 2.7. Data Analysis and Availability

Data and statistical analyses and graphical representations of results were achieved using R (v4.0.3; http://www.r-project.org/) and Prism 9.4 (GraphPad, La Jolla, CA, USA). Mass spectrometry data have been deposited into the ProteomeXchange Consortium (http://proteomecentral.proteomexchange.org) via the PRIDE partner repository with dataset identifier PXD035836.

## 3. Results

### 3.1. Extracorporal Proteome on Hemodialyzer Membranes

**Figure 1:**
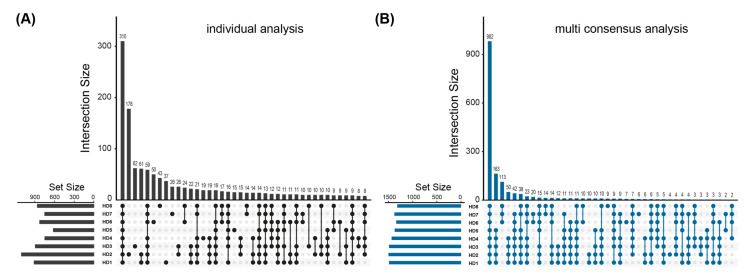
Identification of the hemodialysis membrane core proteome. Upset plots illustrating overlap of identified proteins in each sample. The stacked bars on the left (set size) display the total number of proteins identified in each sample The stacked bar plot at the top shows the number of proteins identified in one or more samples as indicated by the matrix below each bar. (**A**) In total, 310 proteins have been identified in each analyzed sample (core proteome of individual analysis). (**B**) In total, 982 proteins have been identified in each sample with the multi-consensus analysis approach (core proteome of multi-consensus analysis).

To investigate proteins adsorbed onto dialyzer membranes after a single HD session, dialyzers were gently washed of residual blood and then flushed with a strong chaotropic buffer to elute the adsorbed proteins, which were then identified via a mass spectrometric approach. A total of 1648 proteins were identified in eight dialyzers from eight different patients (Supplementary Table S1). Table 1 reports the processed dialyzers 1–8 included in the analysis, indicating the number of identified proteins for the individual and multi-consensus analysis, as well as the dialyzer and membrane type. Of all identified proteins, 310 were found in all samples (Figure 1A). In a multi-consensus analysis of all samples analyzed together, 1736 proteins were identified from the eight dialyzers, 982 of which were found in all samples (Figure 1B). The 310 and 982 proteins found in all samples were considered the HD membrane core proteome in the respective analysis. Quantitative information on the proteins detected was obtained by label-free quantification analysis.

**Figure 2:**
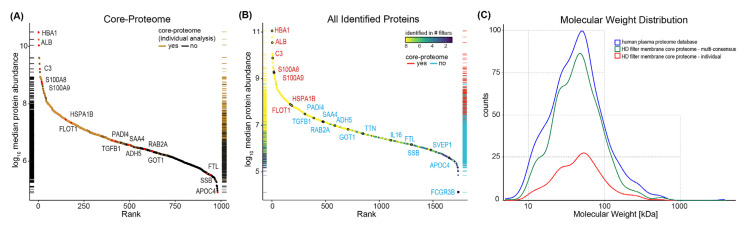
Abundance of proteins adsorbed on hemodialysis membranes. (**A**) Proteins identified in all dialyzers (=core proteome, black circles) in multi-consensus analysis are ranked according to their median abundance. Rug plot on the left side illustrates the protein distribution. Rug plot on the right side displays proteins that are part of the core proteome of the individual analysis (brown). (**B**) All proteins identified in the HD membrane eluates via a multi-consensus analysis are ranked according to median abundance. Rug plots on the left side represent the distribution of the protein abundance and display the number of samples that the proteins have been identified in (color gradient, blue: lowest to yellow: highest). Rug plots on the right side indicate whether proteins have been found in every sample and have therefore been attributed to the core proteome (red). (**C**) Distribution of the molecular weights of identified proteins from the HD membranes (red: individual analysis, green: multi-consensus analysis, blue: reference dataset of the human plasma proteome database) [19].

The ranking of proteins according to their median observed abundance across all samples and by the analysis type (individual vs. multi-consensus) shows that proteins identified in the majority or even all samples of the individual analyses were of higher abundance (Figure 2). Low-abundance proteins were more likely to be identified only in a few or single dialyzers. The HD membrane core proteome of the individual analysis (n = 310) compared to the multi-consensus (n = 982) shows that more abundant proteins are generally identified by individual analysis, while less abundant proteins are more likely to be identified with the multi-consensus approach. The most highly abundant proteins in both analyses are typical plasma proteins, including hemoglobin, albumin and complement component C3. However, this approach is also able to identify proteins bound to the HD membrane with lower plasma abundance, such as interleukin (IL) 16 and small RNA-binding exonuclease protection factor La (SBB). The distribution of the molecular weights of identified proteins from the HD membranes, in comparison to the human plasma proteome, did not show major shifts or selective binding for specific protein sizes (Figure 2C).

### 3.2. Patient-Specific Extracorporeal Proteome

**Figure 3:**
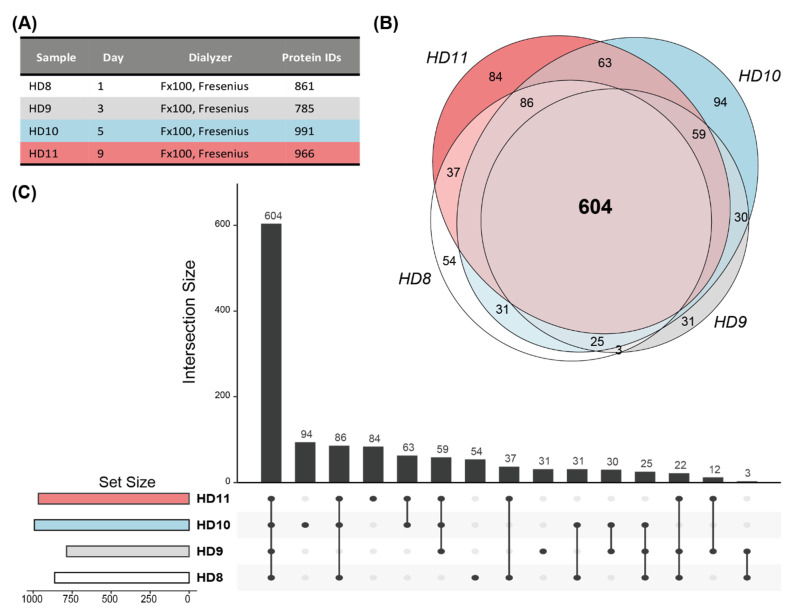
Stability of the patient-specific hemodialysis membrane core proteome. (**A**) Analyzed samples of one patient obtained within 10 days and number of identified proteins in each dialyzer. (**B**) Venn diagram illustrating the overlap of identified proteins per sample (individual analysis). (**C**) Upset plot displaying intersection and set size of identified proteins.

In order to evaluate the stability of our results, we included three more dialyzers from one of the patients that was already included in the analysis, resulting in four longitudinal samples within a time frame of 10 days. This patient was routinely dialyzed using a dialyzer with a helixone membrane (Figure 3A). Individual analysis of the dialyzer resulted in the identification of 1235 unique proteins, of which 604 proteins were found in the common set of all samples (Figure 3B,C). The multi-consensus analysis identified 1180 unique proteins, of which 1088 were detected in all samples (Supplementary Table S2). The analysis of biological replicate samples from the same patient showed that even with the stringent approach of analyzing every dialyzer separately, 50% of the eluate proteome is consistently identified, indicating a very stable patient-specific extracorporeal core proteome. Through the multi-consensus analysis, the number of consistently detected proteins could be boosted to an impressive 92% of all detected proteins.

### 3.3. Enrichment of Cellular Components, Molecular Functions and Molecular Size of the HD Membrane Proteome

**Figure 4:**
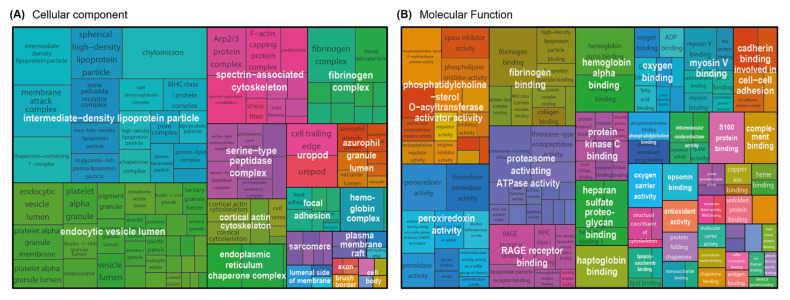
Cellular component and molecular function enrichment analysis of proteins identified from hemodialysis membranes. Gene ontology (GO) cellular component (**A**) and molecular function (**B**) analysis of the core proteome identified (multi-consensus, 982 proteins). Only GO categories with log_2_ fold enrichment >2 were further analyzed with the Revigo tool to summarize terms in a parental term, and the results are visualized as tree maps.

We next characterized the common molecular effects of plasma protein adsorption onto HD membranes, focusing on the core proteome dataset identified in all samples in the multi-consensus analysis. Enrichment analysis of GO cellular component terms was performed to describe the biological connotations of the proteins. The most enriched cellular component terms were related to lipoprotein particles, vesicles, cell membrane and endoplasmic reticulum components and the fibrinogen complex (Figure 4A, Supplementary Table S3). The enrichment analysis of the same proteins for their molecular function revealed the broad and diverse involvement of the proteins bound to hemodialyzer membranes, ranging from fibrinogen and RAGE receptor binding to phosphatidylcholine-sterol and peroxiredoxin activity and to haptoglobin and complement binding (Figure 4B, Supplementary Table S3).

### 3.4. Membrane-Specific Extracorporeal Proteome

**Figure 5:**
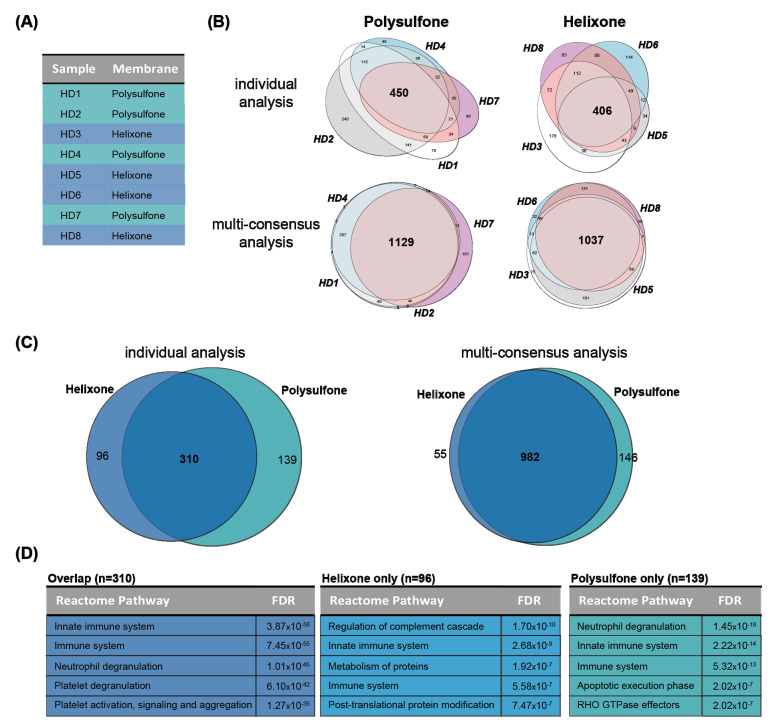
Membrane type-specific differences. (**A**) Analyzed samples and hemodialyzer membrane types. (**B**) Venn diagrams illustrating the overlap of identified proteins in samples with the same membrane type in individual (top) and multi-consensus (bottom) analysis in order to identify the membrane-specific “core proteome”. (**C**) Venn diagram displaying proteins identified exclusively in polysulfone vs. helixone membranes in individual (left) and multi-consensus (right) analysis. (**D**) Reactome pathway enrichment analysis of the three different protein sets (overlap, helixone only, polysulfone only) found in the individual analysis of polysulfone vs. helixone membranes.

Next, we analyzed four modified polysulfone (helixone) membranes and four standard polysulfone membranes from eight patients by individual and multi-consensus analysis (Figure 5A). Each of the analyses identified proteins abundant on the dialyzers with the two membrane types (individual: polysulfone: 449 and helixone: 406 proteins; multiconsensus: polysulfone 1128 and helixone 1037 proteins) (Figure 5B). We then further analyzed overlapping protein sets. In the individual analysis, we found an overlap of 310 proteins identified in both polysulfone and helixone membranes. In total, 96 proteins were found in helixone samples only, and 139 proteins were found in polysulfone samples only, indicating adsorption patterns specific to the membrane type. In the multi-consensus analysis, we found 962 proteins overlapping between both membrane types, and only 55 proteins being helixone- and 146 proteins being polysulfone-specific, respectively (Figure 5C, Supplementary Table S4). Biological pathway enrichment analysis of the 310 proteins found in the individual analysis but on both membrane types enriched immune system and platelet activation-related pathways. The helixone- and polysulfone-specific proteins obtained from the individual analyses showed different enriched biological pathways (Figure 5D). Proteins on both membrane types also led to the enrichment of immune system-related pathways. Proteins only found on polysulfone membranes were additionally enriched for apoptosis and RHO GTPase pathways, while proteins only found on helixone membranes led to the enrichment of pathways related to complement cascade regulation and protein metabolism and modification.

## 4. Discussion

Hemodialysis represents a life-saving therapy for patients with ESKD. Despite its successful application in the clinic, the process of removing excess water and solutes from the patient’s blood is still far from ideal. On the one hand, the removal of so-called protein-bound uremic toxins would be favorable, but the question of how to control the process is still incompletely understood. On the other hand, proteins that are adsorbed to the HD membrane during each dialysis session need to be replenished to avoid malnutrition of these already frail patients. This extracorporeal proteome is a differential plasma proteome, meaning that the difference in plasma between the pre- and post-dialysis situation should correspond to proteins contained in the ultrafiltrate and the proteins adsorbed to the HD membrane [20].

Despite the great potential of used HD membranes as sources of biological insight, without an additional burden on the patient, the available literature on the proteome adsorbed to these membranes is still sparse (Table 2, Supplementary Table S5). In recent years, several proteomic approaches have been evaluated but—due to more recent technological developments—the depth of the proteome analysis was relatively limited [6,9,14,15,21,22,23,24,25]. In a comparison of cellulose triacetate (CTA) dialyzer membranes with helixone dialyzer membranes, it has been shown that both membranes seem to adsorb specific proteins [14]. When high-flux vs. low-flux membranes were compared in two patients, 668 proteins were identified by LC–MS/MS, 177 of which were only retained by high-flux and 320 proteins were only retained by low-flux membranes [26]. In a further study comparing the patterns of proteins adsorbed on recently developed asymmetric cellulose triacetate membranes (ATA), in contrast to conventional CTA membranes, from four chronic HD patients via shotgun LC–MS/MS, a number of proteins ranging from 67 to 130 were identified [9]. Only two studies, published by the same research group, identified more than one hundred unique proteins, but at the cost of lower confidence due to the inclusion of proteins identified on the basis of a single peptide. In our study, we were able to report the largest number of proteins identified from eluted HD membranes in HD patients so far.

The goal of this study was to establish a workflow for the in-depth proteomic characterization of proteins adsorbed to HD membranes. We aimed to assess inter-patient (several patients—one dialyzer each) and intra-patient (one patient—several dialyzers) variability, including an estimate of the effect of the membrane type on the stability of the dialyzer core proteome. Using a workflow based on previously published protocols and tedious optimization experiments, we describe a core proteome, stably identified in all eluates in this study. Two different strategies, varying in their stringency and ability to cover the whole dialyzer proteome, were followed. (i) Individual analysis of raw LC–MS data generated from the different eluates with subsequent matching of confidently identified proteins yielded a list of proteins that are likely to occur in every dialyzer eluate—the evidence from each sample in isolation would have led to the identification of this set of proteins. However, due to the stochastic nature of shotgun proteomics analysis by LC–MS, it is also very likely that this approach misses proteins that are basically found in all samples but may exhibit higher variability or lower overall abundance. (ii) The multi-consensus analysis integrates evidence from the complete set of samples to find matching proteins across samples, whereas, in the individual analysis, every protein has to be identified independently in each sample in order to be available for matching. Through the multi-consensus analysis, the number of proteins consistently detected in dialyzer eluates from the same patient could be boosted to an impressive 92% of all detected proteins, rendering the longitudinal analysis of proteome profiles an option but also leaving some room for more variable signals. We argue that the individual analysis strategy provides highly certain protein identification but may be too stringent for performing a deeper proteome analysis, as there is obviously some evidence that a protein of interest is contained in the HD eluate if it was already identified in one of the other samples. The completeness of the proteomes generated by the individual and the multi-consensus approach was assessed by comparing the molecular weight distributions to the distribution of a consensus plasma resource. Similar peak shapes—and, in the case of the multi-consensus dataset, almost similar numbers—suggest good coverage of the well-established plasma proteome without any obvious size selectivity of the HD membrane-bound fractions.

Although our study focuses on method development and certainly calls for application in larger cohorts with a broader range of membrane materials, this data set may already represent a resource regarding proteins that have been confirmed to be bound on HD membranes. Based on the observation that the core proteome was surprisingly stable over several dialysis sessions in the same patient, we performed several analyses to demonstrate the usefulness of the obtained proteomic data. The analysis of GO terms yielded a broad spectrum of cellular components and molecular functions, clearly leaning towards serum-related terms. Noticeable results were the most prominent terms in both categories being related to lipoproteins, as well as some overrepresented terms, such as RAGE receptor, fibrinogen and vesicle-related terms. Similar to these results, the differential analysis of the influence of membrane materials (polysulfone vs. helixone membrane) on pathway activation was shown to be feasible and yield putatively specifically activated immune pathways. Exploration of the obtained working hypotheses in a larger sample set, however, is obligatory.

Potential further applications of this technology include, for example, multiple myeloma as a typical disease with an expected altered systemic protein pattern and potential need for dialysis [28,29]. Immunologically mediated diseases leading to ESKD are characterized by systemic changes in inflammatory pathways [30]. When blood comes into contact with the dialyzer membrane, activation of the complement system occurs, among other pathways [27]. How this activation of the complement system proceeds in complement-deficient patients has not been elucidated yet. Diabetic nephropathy and vascular mediated nephropathy are among the most common diseases leading to renal failure requiring dialysis. The description of the extracorporeal proteome of patients with the above-mentioned underlying diseases could provide insight into pathomechanistic processes and lead to the identification of new biomarkers.

Limitations of our study are the (still) small sample size, which, in our view, precludes a more general analysis of membrane types or clinical correlates. Moreover, the comprehensive analysis of the extracorporeal proteome of hemodialysis patients would include an analysis of the ultrafiltrate, and potentially the plasma, of these patients to obtain a complete picture. These analyses were not part of this method development but would certainly be part of larger studies demonstrating the full potential of the dialyzer approach.

In conclusion, this study demonstrates the analytical value of used HD membranes regarding coverage of the plasma proteome and potential markers that could be used for monitoring HD therapy and permit a better understanding of the molecular mechanisms of protein loss in patients receiving HD. We show that optimized elution conditions, combined with high-performance proteomics, enable the in-depth exploration of the subproteome bound to the HD membranes of HD patients, yielding a stable core proteome, which, in future studies, can be exploited to study patient-specific factors, develop individualized therapy approaches and aid in the development of novel membrane materials to sustainably improve HD therapy and patient outcomes.

## Figures and Tables

**Table 1:** Processed samples and number of identified proteins in eluate.

Sample #	# Protein ID Multi-Consensus	# Protein ID Individual	Dialyzer	Membrane
1	1493	911	Diacap Pro 19H, Braun	Polysulfone
2	1504	1105	Leoceed, 21HX, Asahi Kasei	Polysulfone
3	1493	892	Fx 100, Fresenius	Helixone
4	1439	747	Leoceed, 21HX, Asahi Kasei	Polysulfone
5	1376	617	Fx 100, Fresenius	Helixone
6	1342	826	Fx100, Fresenius	Helixone
7	1381	749	Leoceed, 21HX, Asahi Kasei	Polysulfone
8	1322	861	Fx100, Fresenius	Helixone

**Table 2:** Previously conducted studies to identify proteins bound on hemodialyzer membranes.

Study	Year	# Filters/Patients	# Proteins Identified	Method to Elute Proteins from Membranes (Wash/Elution Buffer/Elution Procedure)	Proteomic Method	Ref #
Mares et al.	2009	15/5	57 (153 spots)	80 mL 3 mM EDTA 30 min/80 ml 40% acetic acid/30 min circulation (+7000 Da cut-off filter)	2-DGE-MALDI-TOF/TOF	[27]
Urbani et al.	2012	12/6	73	1 L saline/80 mL CSB (6 M urea, 2 M thiourea, 0.4% SDS, 1 mM DTT)/1 h circulation	2-DGE-MALDI-TOF/TOF + LC–MS/MS (DIA)	[14]
Pieroni et al.	2015	6/3	65	1 L saline/80 mL CSB (6 M urea, 2 M thiourea, 0.4% SDS, 1 mM DTT)/1 h circulation	LC–MS/MS (DIA)	[21]
Han et al.	2017	4/2	668 (single-peptide IDs not excluded)	-/1-dodecyl-3-methylimidazolium chloride/ /overnight (+10,000 Da cut-off filter)	dimethyl labeling- LC–MS/MS (DDA)	[26]
Yang et al.	2017	4/4	462 (single-peptide IDs not excluded)	-/1-dodecyl-3-methylimidazolium chloride/ overnight (+10,000 Da cut-off filter)	LC–MS/MS (DDA)	[25]
Ronci et al.	2018	8/4	67–130	3 L saline/180 mL CSB (6 M urea, 2 M thiourea, 0.4% SDS, 1 mM DTT)/1 h circulation	LC–MS/MS (DDA)	[9]
This study	2022	11/8	1648/1736	3 L saline 30 min/125 mL CSB (6 M urea, 2 M thiourea, 0.4% SDS, 1 mM DTT, PI, PPI, pH 8.5)/1 h circulation	LC–MS/MS (DDA)	-

CSB: Chaotropic solubilization buffer, SDS: sodium dodecylsulfate, DTT: dithiothreitol, LC–MS/MS: liquid chromatography–mass spectrometry, DIA: data-independent acquisition, DDA: data-dependent acquisition, 2-DGE: 2-dimensional gel electrophoresis, MALDI: matrix-assisted laser desorption/ionization, TOF: time of flight, PI: proteose inhibitor, PPI: phosphatase inhibitor.

## Data Availability

Mass spectrometry data have been deposited at the ProteomeXchange Consortium (http://proteomecentral.proteomexchange.org) via the PRIDE partner repository with dataset identifier PXD035836.

## Supplementary Materials

The following supporting information can be downloaded at: https://www.mdpi.com/article/10.3390/membranes12111120/s1.
